# An evaluation of high-risk behaviors among female drug users based on Health Belief Model

**Published:** 2015

**Authors:** F Ilika, M Jamshidimanesh, M Hoseini, M Saffari, H Peyravi

**Affiliations:** * School of Nursing and Midwifery, Tehran University of Medical Sciences, Tehran, Iran; ** School of Nursing and Midwifery, Shahroud University of Medical Sciences, Shahroud, Iran,; *** Epidemiology and Biostatistics Department, School of Public Health, Tehran University of Medical Sciences, Tehran, Iran,; **** Education Health Department, Baghiat Allah University, Tehran, Iran,; ***** Center for Nursing Care Research, Iran University of Medical Sciences, Tehran, Iran

**Keywords:** risky behavior, health belief model, drug or substance using women

## Abstract

**Objectives.** Because of the physiological nature of the female reproductive system, women are susceptible to infectious diseases, especially STD and AIDS. Addiction and high-risk behaviors also grow danger of these diseases. The reason of this paper was to examine high-risk behaviors among female drug users based on the Health Belief Model.

**Methods.** Participants of this study were 106 female drug users aged 18 years and older; by the undermost level of literacy skills and been involved in sexual relationships. They came to Drop-In-Centers (DIC) in Tehran, the capital of Iran. Data study was controlled by using a logistic reflux investigation and Pearson correlation analysis.

**Results.** The conclusion showed that women’s overall awareness was moderate. There were a considerable relationship among awareness and years old (p=0.006), awareness and education (p> 0.0001), and awareness and conjugal situation (p=0.062). Perceived sensitivity and severity were clearly compared by education level (p=0.007) and (p=0.014), respectively. Mean scores of perceived benefits and perceived severity of high-risk behaviors were estimated to be superior to other components.

**Conclusion.** Awareness and perceived susceptibility must be raised regarding the educational schedule, which is according to the health belief model in the addiction field, to reduce perceived barriers to risky behavior prevention of women who use drugs.

## Introduction

Drug abuse as a serious global problem would lead to irreparable damages to each society about the personal and common aspects of persons in that society [**1**]. Drug users’ interaction with the environment and their adaptive mechanisms, also their behaviors are psychologically regarded as topics of paramount importance. In some samples, topics are considered as the cause and effect of addiction. High-risk practices are those that make about very unpleasant consequences for addicts or other people [**2**]. Statistics showed that 7.4 percent of the world’s people aged 15 years or older are drug users [**3**]. Iran has the highest per capita rate of heroin and opium addiction in the world. According to the 2006 Census, the number of drug users was estimated at 1.2 million, i.e. about one left of every 2.2 people from the adult population [**4**]. It is estimated that 10 percent of women in Asian countries and 40 percent of them in European countries are drug users. Therefore, the number (16 to 38 million) of female drug abusers in the world is significant [**5**]. 

The United Nations Office on Drugs and Crime (UNODC) reported that 11-21 million people in the world use injectable drugs. Up to 2008, approximately 250,000 injecting drug users had been identified in Iran. Statistics showed that the frequency of including drug usage was 33 percent over the last 30 years and it has had an addition of almost 10 percent over each decade [**6**]. 

It is commonly thought that drug abuse and addiction are primarily attributable to men and that women are less likely to use drugs. Although there is no accurate statistics on the people of female drug users in the country, according to some research, female population include 6.9 percent of addicts of the country. The Ministry of Health also reported that there is one female drug user besides each eight male drug users [**7**]. There is an addition in the likelihood of developing viral infections such as hepatitis B, C, AIDS, among injecting drug users [**8**]. Women make up 3-5 percent of injecting drug users [**5**]. The numbers of female drug users are less than their male counterparts; however, the destructive effects and the severity of addictions as well as the risks are higher for women [**9**]. The results of research carried out in Iran indicated that about 5-17 percent of female drug abusers have unprotected sexual relations [**10**]. Injecting female drug users have a disproportionately higher prevalence of HIV than men [**11**]. Compared to men, sharing syringes and needles is higher for women who live with partners because of the fact that the reachability of injection tools is determined and controlled by their sexual partner [**12**]. Women share needles and syringes with partners who have high-risk sexual behaviors [**13**]. Methamphetamine enhances sexual act, and the use of this material is combined with the use of syringes and needles [**14**]. HIV infection for this population is twice as much as others [**12**]. Because of sharing needles, 2-4 million crowd in advancing zones have been infected with hepatitis C that can be spread to other people. Reviewing the history shows that the prevalence of hepatitis C among injecting drug users ranges from 1.9 to 100% [**15**]. The possibility of women prostitution for the sake of money and drugs raises the chance of HIV disease [**16**]. Tattoos are designed by 35.7 percent of female drug users, and the usage of shared needles to make the operation is calculated at 45 percent of cases [**17**]. A lot of research has revealed that the number of sexually transmitted diseases in injecting and non-injecting drug users are more prevalent, as syphilis is reported to be of 1-6 percent, Chlamydia 1-5 percent, and herpes type II 38-61 percent and HPV types 16 and 18 among female addicts 38 and 42 percent, respectively [**18**]. HBM is one of the original models which uses behavioral science theories for the problems of health-related problems and it is used to explain preventive behaviors [**19**]. This model is comprehensive, further offers to the disease prevention, and explains the correlation among beliefs and behavior. It is founded on the premise that the preventive behaviors are decreasing an individual's vulnerability to diseases and the result of illness on individual lives. They also include hygiene measures in decreasing the hardness of diseases [**20**]. In fact, it investigates the psychological and probable factors affecting the persons’ decisions. Studies have seconded that this model has been proven useful in predicting why people accept or reject various health behaviors. HBM is used as a theoretical framework to study and identify the impact of health beliefs on healthy behaviors [**21**]. The components included in this model contain barriers, noted benefits, perceived awareness, and looked cruelty. These can satisfy the objective of investigating high-risk behaviors associated with female drug users.

**Procedure**

Participants in this cross-sectional study included 106 women with drug-use disorders who came to two Drop-In-Centers (DIC) affiliated to the State Welfare Organization and Family Health Association of Iran in Tehran. 

After obtaining approval from the ethics committee of Medical Science Tehran University along with getting a referral from the health departments and submitting it to the relevant agencies and departments, the researcher thoroughly described the purpose of the research. The selection criteria for female drug users participating in the research were being upper than eighteen years and having sexual relations. After checking the eligibility of participants and having their consent and written permission, they were ensured of the affection of all data introduced. The questionnaire consisted of seven parts as it follows. Data in this questionnaire-based study were collected through spatial databases, books, and articles. The tool consisted of seven parts as it supports: 

*a) Demographic information* containing three parts; personal information with 13 items, 14 items on the past of drug use and sexual behavior background with 16 items, *b) Awareness* (18 items), *c) Perceived sensitivity* of high-risk behaviors (9 items), *d) Perceived severity* of the high-risk behavior consequences (9 items), *e) Perceived benefits* and *Perceived barriers* of preventive behaviors, each containing 6 and 13 items, f). Validity of the application was tested by using content validity. Next, after developing the items, ten faculty members who had expertise and experience in fields of high-risk behaviors, behavior change models in health education, and researching drug users were required to establish the suitability of the questionnaire for the general research and testing hypotheses. Receiving their comments, they were applied and reliability was approved. The safety of the questionnaire was determined through distributing the questionnaires completed by 10 eligible samples. Two weeks later, the same individuals took a retest. It is worth noting that the members in this section of the research were excluded from subsequent phases. The values of Cronbach’ alpha reliability coefficient for awareness was highlighted. The reliability coefficients of perceived sensitivity, perceived severity, perceived benefits, and perceived barriers were also calculated. Finally, based on results obtained through retest and a statistic counselor’s opinion, the reliability of the survey was estimated. The researcher assigned 2 marks for the right results, while evaluating the answers to each item in the awareness section, 1 for incorrect answers, and 0 for an uncertain answer. Other sections of the survey were evaluated based on a five-point Likert Scale (completely agree, agree, neutral, disagree, and completely disagree). Completely agree scored 5 and completely disagree scored 0. For data extraction, information were examined by using SPSS Software version 16 and descriptive/ analytical tests including t-test, logistic regression investigation, and Pearson correlation analysis. 

## Results

Demographic aspects of the 106 participants revealed common age score of 35.7 ± 7.9 years and a standard deviation of the highest frequency of 67% for the age group 30 years and older. A majority of these participants (n=69; 65.1%) had elementary and secondary levels of education. With regard to their marital status, most of them (n= 43; 40.6%) were divorced or widowed (**Table 1**).

**Table 1:** Characteristic descriptive statistics (n=106)

Demographic characteristic		N	%
Age group	20-29	25	24.0
	30-39	45	34.3
	40>	34	32.7
Level of Study	illiterate	5	4.7
	Elementary	69	65.1
	Diploma and above	32	30.2
Marital status	Permanent marriage	29	27.4
	Temporary marriage	31	29.2
	Divorced and Widow	43	40.6
	single	3	5.8

The most common drugs used were glass (81%), opiates (66.7%), heroin (41.9%), methadone (43.8%) and cannabis (26.7%). The most common ways of using drugs were non-injection (85%) and intravenous drug injection (15%). As it was noted, they first experienced drugs used with their husbands (47.1%) and then with their friends (32.1%). 

The mean score observed for women’s awareness with SD ±21.3 was 61 as the lowest and highest scores were 25 and 100, respectively. The scores were split into 3 levels: low, average, and high. Subsequently, the results revealed that maximum of the members (42.1 percent) had moderate awareness about high-risk behaviors. The mean score of perceived sensitivity to high-risk behaviors was 65.2± 17 including the below and highest scores of 28.1 and 100.

Dividing the perceived sensitivity into three groups (namely low, medium, and high), the majority of participants (55.6%) were located in a moderate group. It was in the cause that 61.5 percent of participants obtained a high score regarding the perceived severity of the consequences of high-risk behaviors. That is, the mean score of perceived severity with the lowest and highest scores of 11 and 100, was 76.5 ± 17.2. The base number of the perceived benefits of preventive behaviors with the lowest and highest scores of 25 and 100 was 79.2 ± 19, and 73.6 percent of the members obtained a high score in this section. It is deserving seeing that the highest mean score was received for this section. The bulk of females also recognized small obstacles in performing high-risk behaviors (mean = 44.5± 19.9; Refer to **Table 2**).

**Table 2:** Women's knowledge based on health believe model

Variable	Mean (SD)	Min	Max	Low N (%)	Moderate N (%)	High N (%)
knowledge	61 (21.3)	25.0	100	21 (19.8)	52 (42.1)	33 (31.1)
susceptibility	65.2 (17.)	28.1	100	4 (3.8)	59 (55.6)	43 (40.6)
severity	76.5 (17.2)	11.1	100	3 (2.9)	37 (35.6)	64 (61.5)
benefits	79.2 (19.)	25.0	100	4 (3.8)	24 (22.6)	78 (73.6)
barrier	44/5 (19.9)	0	100	21 (19.8)	52 (42.1)	33 (31.1)

Using the Spearman correlation test, the conclusion revealed that the awareness had a meaningful relationship with age (p=0.006), education (P< 0.0001) and marital status (P=0.062). This means that awareness increased with the increasing in age and degree of study. A meaningful correlation (α= 0.10) was also seen among awareness and conjugal state. Logistic regression investigation was also carried out to simultaneously examine the influence of age, education level, and conjugal state on awareness. It was noted that the marital status had no significant relationship in the appearance of the other 2 variables (**Table 3**).

**Table 3:** Correlation of Knowledge, Aga, Level of Education and Marital Status

		Knowledge				
		Low N (%)	Moderate N(%)	High N(%)	Sum N(%)	P value
Age group	20-29	3 (12.)	10 (40.0)	12 (48.0)	25 (100)	*0.006
	30-39	9 (20.0)	19 (42.2)	17 (37.8)	45 (100)	
	40>	8 (23.5)	22 (64.7)	4 (11.8)	34 (100)	
Level of Education	illiterate	3 (60.0)	2 (40.0)	0 (0)	5 (100)	*<0.0001
	Elementary and Middle school	14 (20.3)	41 (59.4)	14 (20.3)	69 (100)	
	Diploma and Above	4 (12.5)	9 (28.1)	19 (59.4)	32 (100)	
Marital Status	Permanent Marriage	9 (31.0)	11 (37.9)	9 (31.0)	29 (100)	**0.062
	Temporary Marriage	9 (29.0)	14 (45.2)	8 (25.8)	31 (100)	
	Divorced and Widow	3 (7.0)	26 (60.5)	14 (32.6)	43 (100)	
	Single	0 (0)0	1 (33.3)	2 (66.7)	3 (100)	
* Spearman’s Rho Correlation						
** Fisher’s Exact						

Perceived sensitivity was not significantly correlated with age and conjugal state; however, it had a meaningful relationship with the education level (P=0.007). The greater the study step was, the famous the perceived sensitivity. Perceived severity and benefits had no meaningful correlation with age, study level, or marital status. It was in the case that a meaningful correlation was seen among recognized obstacles and education levels (p=0.014) (**Table 4**,**5**). 

**Table 4:** Correlation of Perceived Susceptibility, Perceived Severity, Aga, level of Education and Marital Status

		Perceived Susceptibility					Perceived Severity				
Demographic Characteristics		Low N (%)	Moderate N(%)	High N (%)	Sum N (%)	P value	Low N (%)	Moderate N (%)	High N (%)	Sum N (%)	P value
Age group	20-29	1 (4.0)	11 (44.0)	13 (52.0)	25 (100)	*0.735	1 (4.2)	9 (37.5)	14 (58.3)	24 (100)	*0.752
	30-39	1 (2.2)	29 (64.4)	15 (33.3)	45 (100)		0 (0)	18 (40.0)	27 (60.0)	45 (100)	
	40>	1 (2.9)	18 (52.9)	15 (44.1)	34 (100)		2 (6.1)	10 (30.3)	21 (63.6)	33 (100)	
Level of Education	illiterate	1 (20.0)	3 (60.0)	1 (20.0)	5 (100)	*0.007	0 (0)	1 (20.0)	4 (80.0)	5 (100)	*0.484
	Elementary and Middle school	2 (2.9)	44 (63.8)	23 (33.3)	69 (100)		2 (2.9)	24 (35.3)	42 (61.8)	68 (100)	
	Diploma and Above	1 (3.1)	12 (37.5)	19 (59.4)	32 (100)		1 (3.2)	12 (38.7)	18 (58.1)	31 (100)	
Marital Status	Permanent Marriage	1 (3.4)	17 (58.6)	11 (37.9)	29 (100)	**0.728	0 (0)	11 (37.9)	18 (62.1)	29 (100)	**0.781
	Temporary Marriage	0	19 (61.3)	12 (38.7)	31 (100)		2 (6.7)	11 (36.7)	17 (56.7)	30 (100)	
	Divorced and Widow	3 (7.0)	22 (51.2)	18 (41.9)	43 (100)		1 (2.3)	15 (34.9)	27 (62.8)	43 (100)	
	Single	0	1 (33.3)	2 (66.7)	3 (100)		0 (0)	0 (0)	2 (100)	2 (100)	
* Spearman’s Rho Correlation ** Fisher’s Exact Test											

**Table 5:** Correlation of Perceived Benefits, Perceived Barriers, Aga, level of Education and Marital Status

		Perceived Benefits					Perceived Barriers				
Demographic Characteristics		Low N (%)	Moderate N(%)	High N (%)	Sum N (%)	P value	Low N (%)	Moderate N (%)	High N (%)	Sum N (%)	P value
Age group	20-29	1 (4.0)	10 (40.0)	14 (56.0)	25 (100)	*0.313	6 (24)	18 (72.0)	1 (40)	25 (100)	*/0.233
	30-39	0 (0)	7 (15.9)	38 (84.4)	45 (100)		16 (35.6)	23 (51.1)	6 (13.3)	45 (100)	
	40>	3 (8.8)	6 (17.6)	25 (73.5)	34 (100)		16 (47.1)	14 (41.2)	4 (11.8)	34 (100)	
Level of Education	illiterate	0 (0)	1 (20.0)	4 (80.0)	5 (100)	*0.467	4 (80.0)	1 (20.0)	0 (0)	5 (100)	*0.014
	Elementary and Middle school	3 (4.3)	14 (20.3)	52 (75.4)	69 (100)		29 (42.0)	33 (47.8)	7 (10.1)	69 (100)	
	Diploma and Above	1 (3.1)	9 (28.1)	22 (68.8)	32 (100)		7 (21.9)	21 (65.6)	4 (12.5)	32 (100)	
Marital Status	Permanent Marriage	0 (0)	6 (20.7)	23 (79.3)	29 (100)	**0.562	12 (41.4)	14 (48.3)	3 (10.3)	29 (100)	**0.904
	Temporary Marriage	1 (3.2)	10 (32.3)	20 (64.5)	31 (100)		9 (29.0)	19 (61.3)	3 (9.7)	31 (100)	
* Spearman’s Rho Correlation ** Fisher’s Exact Test											

## Discussion 

The results obtained revealed that the common age score of participants was 35.7 ± 7.9 years. Also, the general age of 34.5 ±11.2 years was reported, which is in position with that of the present study. In this study, the main drugs used were reported first to be glass and then methadone and heroin, and hashish. The reported order of these commonly used drugs was as it follows: opium, crack, glass, ecstasy, and heroin [**22**]. Comparing the results, it can be concluded that differences in drug users’ attitudes are because that glass is of lower cost and easily accessible. Most participants had first experienced using drugs with their husbands. They stated that men shared drugs with their wives to avoid their objections and to have a companion in using drugs.

The base number of women’s awareness of high-risk behaviors was moderate. Behaviors such as tattooing (49.5%) and loss condom use in each 10 sexual relationships (45.9%) were also reported across the least 3 months. The experience of shared use of syringes and needles and symptoms of sexually spread infections were respectively reported; 17.9% and 34% during the past three months. Karimi’s research on male drug users indicated that 52.8 percent of them had a high awareness, while 39.5 percent of them did not perform well and had not taken preventive measures. They also had a background of high-risk behaviors such as unprotected sex and drug injection [**3**].

The two studies reviewed above pointed to a conclusion that women are less aware than men, perhaps due to gender differences, lower employment, lower education levels, and less social interactions. Women’s awareness step in the modern research was significantly correlated with age, education level, and conjugal state. Increasing age was also compared with upper awareness in the Sabooteh’s study [**23**]. In this study, women having diploma or higher education levels were more cognizant. It is clear that people with upper learning levels possess higher awareness levels and observe themselves more susceptible to damages. Education and awareness play a fundamental part in maintaining health. Illiteracy can cause lack of responsibility for health and treatments issues [**24**]. Another research discovered that teenagers with higher steps of study were more cognizant and saw themselves as more vulnerable; however, adolescents with top steps of study are extra suitable than others to finance more risky sexual behavior [**25**]. Age mostly affects high-risk practices so happen in youth. Tenkorang claimed that age is a predictor of high-risk behaviors, especially sexual behavior in adolescents since people do not see themselves exposed to major risks such as AIDS and are extra suitable to attempt risky behavior [**25**]. In his study, Hanton found no link between awareness and education/ age, whereas a high steps of information in current research was reported. This reflects the young age of the participants ranging 15- 24 years [**26**]. 

In the current research, the HBM constructs of perceived sensitivity to high-risk behaviors had a meaningful correlation by education level. However, there was no meaningful correlation among perceived sensitivity and age/ marital status. The current research is in consensus with Solhi’s [**24**]. Perceived sensitivity has a strong cognitive component and is somewhat dependent on individual knowledge [**27**].

It can be assumed that more sensitivity is probably due to training classes in drop-in centers and shelters or to regular and periodic examinations to detect new cases of HIV and hepatitis. Rahmati also obtained the same results based on the above-mentioned model. The researcher introduced media as its cause [**20**]. In studies conducted by Vakili, Aser, and Soldi, constructs of perceived sensitivity were met in a lowest rate. It is perhaps because the members in this research saw themselves at no risk. For example, women who played in Vakili’s study served as health liaisons. Monogamous women participating in Aser’s study ignored the chance of HIV disease. Participants of Solhi’s study included barbers who did not know themselves susceptible to hepatitis and AIDS [**21**,**24**,**28**].

Participants who are short tender to being infected with HIV are more susceptible. This reduced sensitivity leads to a decrease in accuracy of prudent behaviors and exposes many people to the risk of HIV/ AIDS as well [**29**]. Perceived sensitivity has the prime role in understanding the behavior. If a person is sensitive to health problems and recognizes that symptoms cannot only be due to certain diseases, this sensitivity then leads to the prevention of high-risk behavior and HIV infection [**24**]. High-perceived sensitivity is necessary to improve the motive of participants in preventive health behaviors [**21**]. Compared to men, women considered themselves more susceptible to AIDS and would choose protective behavior such as using condom and having fewer sexual partners [**26**]. The perceived sharpness of the existing research was high. It seems that people perceive diseases such as AIDS and Hepatitis as diseases with severe consequences and consider themselves at risk. This is because the women participating in our study observed the risk of illness or death in their family due to AIDS and hepatitis. 

Tenkorang mentioned that experiences and consequences surrounding the death of families could have a extra real influence on perceived severity and feeling higher risk than others [**25**]. Like sensitivity, severity also has a strong cognitive component and is dependent upon individual knowledge [**27**]. The results showed that age, education level, and marital state has no meaningful connection with perceived severity. These results are in a similar vein with other studies [**20**,**24**,**30**]. A high score means that the variables are not associated with perceived severity. Tenkorang, albeit, found a correlation between education level and perceived severity and 62.5% of the members in his study with middle school study level perceived no risk of high-risk behaviors. He also mentioned that the high education level is associated with the rejection of traditional and religious teachings [**25**]. Zack found out that students have had little perceived severity of STD and have taken less preventive behaviors about it [**31**], which may be owing to the above reason. The perceived benefits of the current research were high and the highest mean score was related to this component. 

It can be concluded that members who received a service in these centers took preventive actions. Perceived benefits had no meaningful correlation with each of the changeable (namely age, education level, and conjugal state). Vakili argued that fairly individuals’ high planes of observed benefits represent their knowledge of the possible preventive behaviors [**21**]. Lin reported that the above-mentioned perceived benefits about one preventive factor lead to performing more preventive behaviors than other constructs [**32**]. 

According to the health belief model, when an individual reaches an appropriate understanding level of beliefs about sensitivity and severity, he does not accept health recommendations unless the potential benefits versus the obstacles of that behavior are well-understood [**30**].

Aser et al. introduced perceived benefits of condom use as the most significant structural characters of the health belief model. This research revealed that the correlation among benefits and the usage condoms exist as a preventive behavior [**28**]. The majority of women in this research scored medium for perceived barriers. Perceived difficulties had no important correlation including age and marital status, while they had an important association with the education level. Perhaps, it is because of paying more regard to removing barriers in adopting a behavior. Perceived barriers play a vital role in predicting protective health behaviors [**3**]. Namdar also confirmed a significant relationship between perceived barriers and education level of women aged 20-65 years [**33**]. Volk’s findings indicated that perceived barriers among men and women are only a part of the Health Belief Model and are effective on the behavior of condom use [**34**]. People who perceived fewer barriers have more preventive behaviors [**32**]. Zaho et al. showed that perceived barriers to condom use in prostitutes were more than the perceived benefits. Thus, reducing barriers to condom use is more effective than raising awareness [**35**].

## Conclusion 

Because of the experience that the high perceived sensitivity to enhance the motivation of individuals to adopt preventive health behaviors and that perceived benefits is the most significant structural characters of the Health Belief Model and that awareness and perceived barriers are predicted to value for strong acquired behaviors, and it seems that educational planning should be provided and executed based on behavior change models like the HBM to prevent high-risk behaviors in this group of vulnerable women and to decrease barriers and increase their awareness of other structures and models. One of the restrictions of this research is the participants' self-reporting. The goal was set up to reach the highest accurate data from the participants through their ensuring of the secret of the questionnaires and gift giving. Another restrictions is that the participants of this research were just those women coming to drop-in centers. Result shows, the conclusion of this research is restricted to those women not coming to these centers, attending drug rehab camps, and homeless women. Hence, mobile treatment teams are needed to investigate their high-risk behaviors.

**Ethical considerations**

The research was allowed by the standards board of the Iran University. The researcher obtained permission of the participants. All women were informed regarding the confidential quality of the information. All participants were educated that they would be voluntary in refusing to response any questions. All participants were educated that they would be free to withdraw from the research at each time. All members were encouraged to ask any questions or concerns about their participation.

**Acknowledgement**

Author would like to thank all females who associated in the research.

**Funding Support**

The research was funded with Research Vice Chancellor of Medical Science Tehran University and Center for Nursing Care Research, Iran Medical Science Tehran University. We kindly express our appreciation to Tehran and Iran Medical Science Tehran University.
